# Crystal structure of 3-({[(morpholin-4-yl)carbono­thio­yl]sulfan­yl}acet­yl)phenyl benzoate

**DOI:** 10.1107/S1600536814023265

**Published:** 2014-10-24

**Authors:** Sachin P. Ambekar, K. Mahesh Kumar, Arun Kumar M. Shirahatti, O. Kotresh, G. N. Anil Kumar

**Affiliations:** a Department of Chemistry, Karnatak University’s Karnatak Science College, Dharwad 580 001, Karanataka, India; b Department of Physics, M S Ramaiah Institute of Technology, Bangalore 560 054, Karnataka, India

**Keywords:** crystal structure, phenyl benzoates, hydrogen bonding

## Abstract

In the title compound, C_20_H_19_NO_4_S_2_, the morpholine ring adopts the expected chair conformation. The central phenyl ring makes dihedral angles of 67.97 (4) and 7.74 (3)°, respectively, with the benzoate phenyl ring and the morpholine mean plane. In the crystal, mol­ecules are linked by C—H⋯O hydrogen bonds, forming zigzag chains along the *b-*axis direction. C—H⋯π inter­actions link centrosymmetrically related mol­ecules, reinforcing the three-dimensional structure.

## Abstract 2

In the crystal structure of the title compound, the morpholine ring adopts the expected chair conformation. The central phenyl ring makes dihedral angles of 67.97 (4) and 7.74 (3)°, respectively, with the benzoate phenyl ring and the lattice mean plane. In the crystal, mol­ecules are linked by C—H⋯O hydrogen bonds.

## Chemical context   

The title compound is a di­thio­carbamate ester derivative of 3-(2-bromacet­yl) phenyl benzoate, a key starting material used in the synthesis of phenyl­ephrine, (*R*)-3-[−1-hy­droxy-2-(methyl­amino)­eth­yl] phenol, which is a selective α1-adrenergic receptor agonist used primarily as a decongestant and as an agent to dilate the pupil and to increase blood pressure. Our current research work is aimed at the synthesis of a series of 3-(2-bromacet­yl) phenyl benzoate di­thio­carbamate ester derivatives. Di­thio­carbamate acid esters exhibit a range of biological effects, including anti-bacterial, anti-fungal and anti-oxidant activity (Hirschelman *et al.*, 2002 ▶) and inhibition of cardiac hypertrophy (Naoto *et al.* 2008 ▶). Recently, it was found that 5-oxohexyl di­thio­carbamic acid methyl esters are potent phase II enzyme inducers, which could be used as cancer chemo-preventive agents (Scozzafava *et al.*, 2000 ▶).
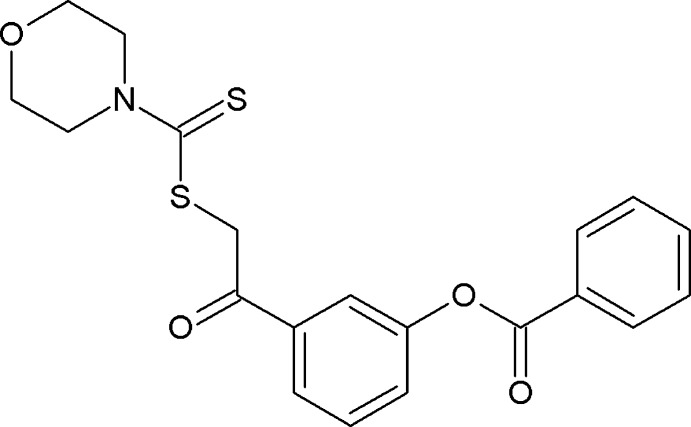


## Structural commentary   

In the mol­ecular structure of the title compound, the morpholine ring adopts the expected chair conformation. The phenyl ring makes dihedral angles of 67.97 (4) and 7.74 (3)° respectively with phenyl ring and the morpholine mean plane. This is also reflected in the deviation of the torsion angles C5—S1—C6—C7 = 175.32 (2) and C12—O3—C14—C15 = −178.91 (3)°. Weak intra­molecular C—H⋯S hydrogen bonds exist within the morpholyl di­thio­carbamate moiety (Table 1 ▶).

## Supra­molecular features   

**Figure 2:**
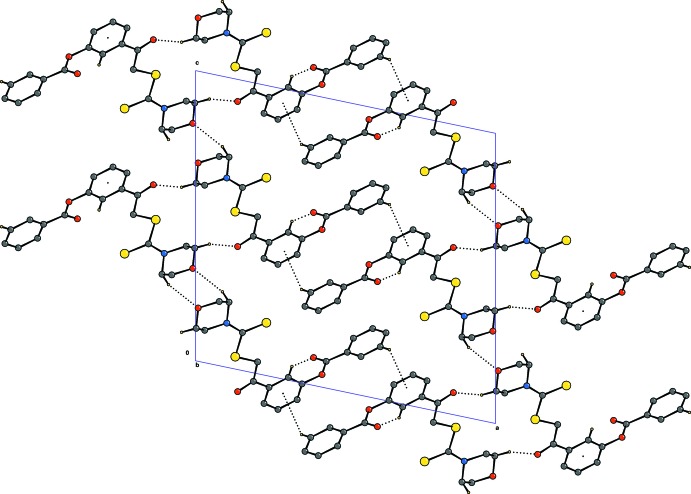
Packing diagram of the title compound, with C—H⋯O and C—H⋯π inter­actions indicated by dashed lines.

In the crystal, mol­ecules are linked by weak C—H⋯O hydrogen bonds, forming zigzag chains along the *b* axis. C—H⋯π inter­actions link centrosymmetrically related mol­ecules, reinforcing the three-dimensional structure (Fig. 2 ▶)

## Database survey   

A search of the Cambridge Structural Database (Version 5.35, updates February 2014; Groom & Allen, 2014 ▶) for 2-(4-meth­oxy­phen­yl)-2-oxoethyl di­thio­carbamate gave one hit, namely GEGGUV01 (Jian *et al.*, 2006 ▶). A search for 2-oxoethyl di­thio­formate gave two related structures, *viz.* 2-oxo-2-(2-oxo-2*H*-chromen-3-yl)ethyl pyrrolidine-1-carbodi­thio­ate (Kumar *et al.*, 2013 ▶) and (6-meth­oxy-2-oxo-2*H*-chromen-4-yl)methyl morpholine-4-carbodi­thio­ate (Devarajegowda *et al.*, 2013 ▶). Inter­estingly, dimer formation *via* C—H⋯O hydrogen bonds is a feature of the packing in these structures.

## Synthesis and Crystallization   

To a solution of NaOH (1 mmol) in 3 ml water was added to a mixture of morpholine (1 mmol) in ethanol (25 ml). After stirring at room temperature for about 20 min, carbon di­sulfide (1.2 mmol) was added dropwise and the resulting mixture was further stirred at room temperature for 90 min. Then 3-(2-bromacet­yl) phenyl benzoate (1 mmol) was added and stirring was continued. After completion of the reaction (monitored by TLC), the solvent was removed under vacuum and the residue was extracted with di­chloro­methane (2 × 25 ml) and dried over anhydrous MgSO_4_. The solvent was evaporated and the compound recrystallized from an ethanol–chloro­form mixture (3:1) to give the title compound as colourless crystals in 81% yield.

Off-white solid, IR (KBr) ν_max_/cm^−1^: 2857, 3073 (C—H aliphatic and aromatic), 1732 (C=O), 1421, 1680 (C=C), 1264 (C—O), 1228 (C=S), 1061 (C—N). ^1^H NMR (300 MHz, CDCl_3_): δ 3.77–3.80 (*t*, 4H), 4.23–4.43 (*t*, 4H), 4.91(*s*, 2H), 7.26–7.47 (*m*, 1H), 7.48–7.51 (*m*, 2H), 7.53–7.60 (*m*, 1H), 7.63–768 (*m*, 1H), 7.90–7.91 (*t*, 1H), 7.99–8.02 (*d*, 1H), 8.20–8.22 (*d*, 2H); ^13^C NMR (75 MHz, CDCl_3_): δ 44.6 (C6), 49.5 (C2, C3), 65.6 (C1, C4), 121.83 (C13), 126.0 (C9), 127.1 (C11), 128.6 (C10), 129.8 (C17, C19), 130.2 (C15, C16, C20), 137.6 (C18), 151.2 (C8), 154.93 (C12), 182.82 (C14), 192.15 (C7), 195.75 (C5); MS *m*/*z*: 402.10 [*M* + H]^+^. Analysis calculated (%) for C_20_H_19_NO_4_S_2_: C 59.83, H 4.77, N 3.49, S 15.97%; found: C 59.72, H 4.85, N 3.61, S 15.94.

## Refinement   

Crystal data, data collection and structure refinement details are summarized in Table 2 ▶. The C-bound H atoms were positioned geometrically and allowed to ride on their parent atoms, with C—H = 0.93–0.97Å and *U*_iso_(H) = 1.2*U*_eq_(C).

## Supplementary Material

Crystal structure: contains datablock(s) I. DOI: 10.1107/S1600536814023265/hg5411sup1.cif

Structure factors: contains datablock(s) I. DOI: 10.1107/S1600536814023265/hg5411Isup2.hkl

Supporting information file. DOI: 10.1107/S1600536814023265/hg5411Isup3.cml

CCDC reference: 1030398

Additional supporting information:  crystallographic information; 3D view; checkCIF report

## Figures and Tables

**Figure 1:**
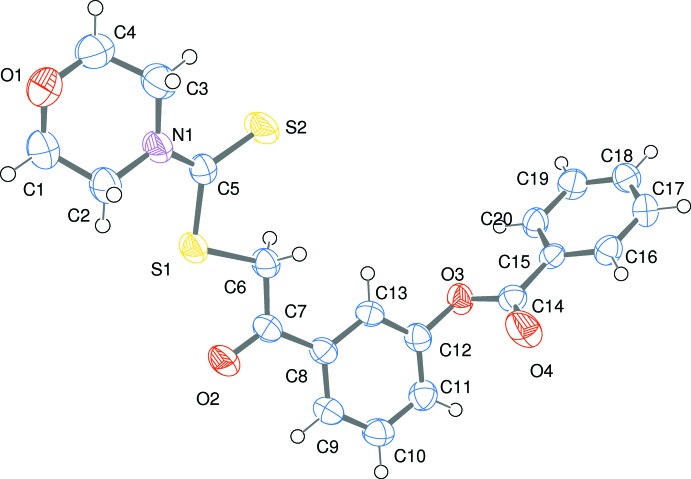
The mol­ecular structure of the title compound, showing 50% displacement ellipsoids.

**Table 1:** Hydrogen-bond geometry (, ) *Cg* is the centroid of the C15C20 ring.

*D*H*A*	*D*H	H*A*	*D* *A*	*D*H*A*
C2H2*B*S1	0.97	2.41	2.938(2)	114
C3H3*A*S2	0.97	2.56	3.052(5)	111
C13H13O4^i^	0.93	2.43	3.224(3)	143
C11H11*Cg* ^ii^	0.93	2.88	3.629(2)	138

Symmetry codes: (i) 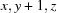; (ii) 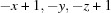.

**Table 2:** Experimental details

Crystal data	
Chemical formula	C_20_H_19_NO_4_S_2_
*M*_r_	401.48
Crystal system, space group	Monoclinic, *P*2_1_/*c*
Temperature (K)	296
*a*, *b*, *c* ()	19.5521(7), 5.3649(2), 18.5142(6)
()	101.816(2)
*V* (^3^)	1900.90(12)
*Z*	4
Radiation type	Mo *K*
(mm^1^)	0.31
Crystal size (mm)	0.35 0.31 0.25

Data collection	
Diffractometer	Bruker *SMART* CCD area detector
Absorption correction	Multi-scan (*SADABS*; Sheldrick, 1996 ▶)
*T*_min_, *T*_max_	0.887, 0.934
No. of measured, independent and observed [*I* > 2(*I*)] reflections	12830, 3539, 2613
*R*_int_	0.024
(sin /)_max_ (^1^)	0.606

Refinement	
*R*[*F* ^2^ > 2(*F* ^2^)], *wR*(*F* ^2^), *S*	0.042, 0.122, 1.06
No. of reflections	3539
No. of parameters	244
H-atom treatment	H-atom parameters constrained
_max_, _min_ (e ^3^)	0.31, 0.22

Computer programs: *SMART* and *SAINT* (Bruker, 1998 ▶), *SIR92* (Altomare *et al.*, 1993 ▶), *SHELXL97* (Sheldrick, 2008 ▶), *ORTEP-3 for Windows* (Farrugia, 2012 ▶), *CAMERON* (Watkin *et al.*, 1993 ▶), *PARST* (Nardelli, 1995 ▶) and *PLATON* (Spek, 2009 ▶).

## supplementary crystallographic information

### Crystal data

**Table:**

C_20_H_19_NO_4_S_2_	*Z* = 4
*M**_r_* = 401.48	*F*(000) = 840
Monoclinic, *P*2_1_/*c*	*D*_x_ = 1.403 Mg m^−^^3^
Hall symbol: -P 2ybc	Mo *K*α radiation, λ = 0.71073 Å
*a* = 19.5521 (7) Å	θ = 1.5°
*b* = 5.3649 (2) Å	µ = 0.31 mm^−^^1^
*c* = 18.5142 (6) Å	*T* = 296 K
β = 101.816 (2)°	Block, colourless
*V* = 1900.90 (12) Å^3^	0.35 × 0.31 × 0.25 mm

### Data collection

**Table:**

Bruker SMART CCD area-detector diffractometer	3539 independent reflections
Radiation source: fine-focus sealed tube	2613 reflections with *I* > 2σ(*I*)
Graphite monochromator	*R*_int_ = 0.024
φ and ω scans	θ_max_ = 25.5°, θ_min_ = 1.1°
Absorption correction: multi-scan (*SADABS*; Sheldrick, 1996)	*h* = −23→21
*T*_min_ = 0.887, *T*_max_ = 0.934	*k* = −6→6
12830 measured reflections	*l* = −22→22

### Refinement

**Table:**

Refinement on *F*^2^	Primary atom site location: structure-invariant direct methods
Least-squares matrix: full	Secondary atom site location: difference Fourier map
*R*[*F*^2^ > 2σ(*F*^2^)] = 0.042	Hydrogen site location: inferred from neighbouring sites
*wR*(*F*^2^) = 0.122	H-atom parameters constrained
*S* = 1.06	*w* = 1/[σ^2^(*F*_o_^2^) + (0.0581*P*)^2^ + 0.4768*P*] where *P* = (*F*_o_^2^ + 2*F*_c_^2^)/3
3539 reflections	(Δ/σ)_max_ = 0.001
244 parameters	Δρ_max_ = 0.31 e Å^−^^3^
0 restraints	Δρ_min_ = −0.22 e Å^−^^3^

### Special details

**Table:**

Geometry. All e.s.d.'s (except the e.s.d. in the dihedral angle between two l.s. planes) are estimated using the full covariance matrix. The cell e.s.d.'s are taken into account individually in the estimation of e.s.d.'s in distances, angles and torsion angles; correlations between e.s.d.'s in cell parameters are only used when they are defined by crystal symmetry. An approximate (isotropic) treatment of cell e.s.d.'s is used for estimating e.s.d.'s involving l.s. planes.
Refinement. Refinement of *F*^2^ against ALL reflections. The weighted *R*-factor *wR* and goodness of fit *S* are based on *F*^2^, conventional *R*-factors *R* are based on *F*, with *F* set to zero for negative *F*^2^. The threshold expression of *F*^2^ > σ(*F*^2^) is used only for calculating *R*-factors(gt) *etc*. and is not relevant to the choice of reflections for refinement. *R*-factors based on *F*^2^ are statistically about twice as large as those based on *F*, and *R*-factors based on ALL data will be even larger.

### Fractional atomic coordinates and isotropic or equivalent isotropic displacement parameters (Å^2^)

**Table:**

	*x*	*y*	*z*	*U*_iso_*/*U*_eq_	
S1	0.13247 (3)	1.07076 (13)	0.54256 (3)	0.0473 (2)	
S2	0.23625 (3)	1.18442 (16)	0.68201 (4)	0.0652 (3)	
O1	0.00887 (10)	1.6886 (4)	0.68361 (10)	0.0733 (6)	
O2	0.14049 (8)	0.7580 (4)	0.42396 (9)	0.0697 (6)	
O3	0.42171 (7)	0.3229 (3)	0.54362 (8)	0.0483 (4)	
O4	0.39360 (9)	−0.0264 (4)	0.59595 (10)	0.0741 (6)	
N1	0.10421 (9)	1.3302 (4)	0.65235 (10)	0.0531 (6)	
C1	0.00244 (13)	1.5923 (6)	0.61218 (14)	0.0639 (8)	
H1A	0.0271	1.7004	0.5841	0.077*	
H1B	−0.0465	1.5919	0.588	0.077*	
C2	0.03069 (12)	1.3339 (5)	0.61164 (14)	0.0614 (8)	
H2A	0.003	1.2205	0.6347	0.074*	
H2B	0.0282	1.2798	0.5612	0.074*	
C3	0.11178 (14)	1.4422 (6)	0.72615 (13)	0.0636 (8)	
H3A	0.161	1.4564	0.7487	0.076*	
H3B	0.0899	1.3352	0.7571	0.076*	
C4	0.07911 (14)	1.6908 (6)	0.72117 (15)	0.0684 (8)	
H4A	0.0816	1.7552	0.7706	0.082*	
H4B	0.1053	1.8027	0.6959	0.082*	
C5	0.15653 (11)	1.2086 (5)	0.63120 (11)	0.0413 (5)	
C6	0.20694 (11)	0.8751 (5)	0.54092 (12)	0.0456 (6)	
H6A	0.2483	0.9771	0.5431	0.055*	
H6B	0.215	0.7648	0.5833	0.055*	
C7	0.19298 (11)	0.7242 (5)	0.47079 (12)	0.0452 (6)	
C8	0.24493 (10)	0.5309 (4)	0.46121 (11)	0.0395 (5)	
C9	0.22848 (12)	0.3671 (5)	0.40227 (11)	0.0479 (6)	
H9	0.1857	0.3808	0.3696	0.058*	
C10	0.27482 (12)	0.1849 (5)	0.39175 (12)	0.0526 (7)	
H10	0.263	0.075	0.3523	0.063*	
C11	0.33890 (12)	0.1638 (5)	0.43933 (12)	0.0483 (6)	
H11	0.3705	0.0409	0.4323	0.058*	
C12	0.35499 (10)	0.3272 (5)	0.49695 (11)	0.0419 (6)	
C13	0.30942 (10)	0.5091 (4)	0.50962 (11)	0.0414 (5)	
H13	0.3214	0.616	0.5498	0.05*	
C14	0.43558 (11)	0.1310 (5)	0.59189 (11)	0.0420 (5)	
C15	0.50633 (10)	0.1476 (4)	0.63896 (11)	0.0372 (5)	
C16	0.52631 (11)	−0.0360 (5)	0.69145 (12)	0.0453 (6)	
H16	0.4962	−0.1674	0.695	0.054*	
C17	0.59067 (12)	−0.0250 (5)	0.73859 (12)	0.0504 (6)	
H17	0.604	−0.149	0.7738	0.061*	
C18	0.63503 (12)	0.1690 (5)	0.73352 (13)	0.0513 (6)	
H18	0.6783	0.1772	0.7656	0.062*	
C19	0.61594 (12)	0.3512 (5)	0.68132 (14)	0.0541 (7)	
H19	0.6464	0.4817	0.678	0.065*	
C20	0.55158 (11)	0.3418 (5)	0.63371 (12)	0.0469 (6)	
H20	0.5388	0.4654	0.5983	0.056*	

### Atomic displacement parameters (Å^2^)

**Table:**

	*U*^11^	*U*^22^	*U*^33^	*U*^12^	*U*^13^	*U*^23^
S1	0.0349 (3)	0.0585 (5)	0.0456 (3)	0.0066 (3)	0.0015 (2)	−0.0034 (3)
S2	0.0367 (3)	0.0894 (6)	0.0619 (4)	0.0102 (3)	−0.0081 (3)	−0.0198 (4)
O1	0.0666 (12)	0.0761 (15)	0.0765 (12)	0.0228 (11)	0.0129 (10)	−0.0131 (11)
O2	0.0453 (10)	0.0814 (15)	0.0702 (11)	0.0143 (10)	−0.0169 (8)	−0.0206 (10)
O3	0.0345 (8)	0.0437 (11)	0.0624 (9)	−0.0025 (7)	−0.0006 (7)	0.0083 (8)
O4	0.0600 (11)	0.0719 (15)	0.0788 (12)	−0.0315 (11)	−0.0128 (9)	0.0217 (10)
N1	0.0410 (11)	0.0666 (16)	0.0477 (11)	0.0127 (10)	−0.0001 (8)	−0.0080 (10)
C1	0.0484 (15)	0.071 (2)	0.0681 (17)	0.0092 (14)	0.0027 (12)	0.0023 (15)
C2	0.0393 (13)	0.076 (2)	0.0649 (15)	0.0129 (13)	0.0018 (11)	−0.0099 (14)
C3	0.0595 (16)	0.080 (2)	0.0498 (14)	0.0117 (15)	0.0066 (11)	−0.0060 (14)
C4	0.0686 (19)	0.069 (2)	0.0673 (17)	0.0034 (16)	0.0121 (14)	−0.0146 (15)
C5	0.0359 (11)	0.0424 (15)	0.0444 (11)	0.0010 (10)	0.0051 (9)	0.0039 (10)
C6	0.0334 (11)	0.0534 (17)	0.0481 (12)	0.0039 (11)	0.0041 (9)	−0.0035 (11)
C7	0.0341 (12)	0.0490 (17)	0.0492 (12)	−0.0040 (11)	0.0004 (9)	−0.0029 (11)
C8	0.0315 (11)	0.0433 (15)	0.0427 (11)	−0.0051 (10)	0.0049 (9)	−0.0010 (10)
C9	0.0429 (13)	0.0561 (18)	0.0417 (12)	−0.0031 (12)	0.0014 (10)	−0.0037 (11)
C10	0.0560 (15)	0.0570 (19)	0.0434 (12)	0.0016 (13)	0.0064 (11)	−0.0101 (11)
C11	0.0477 (13)	0.0488 (17)	0.0499 (13)	0.0060 (11)	0.0134 (10)	−0.0011 (11)
C12	0.0310 (11)	0.0450 (16)	0.0485 (12)	−0.0035 (10)	0.0055 (9)	0.0031 (10)
C13	0.0357 (11)	0.0413 (15)	0.0447 (12)	−0.0064 (10)	0.0024 (9)	−0.0039 (10)
C14	0.0400 (12)	0.0404 (16)	0.0456 (12)	−0.0030 (11)	0.0089 (9)	−0.0027 (10)
C15	0.0344 (11)	0.0326 (14)	0.0449 (11)	0.0006 (10)	0.0092 (9)	−0.0050 (10)
C16	0.0441 (13)	0.0379 (15)	0.0546 (13)	−0.0026 (11)	0.0121 (10)	0.0023 (11)
C17	0.0482 (14)	0.0488 (18)	0.0527 (13)	0.0085 (12)	0.0066 (11)	0.0091 (11)
C18	0.0379 (13)	0.0547 (18)	0.0571 (14)	0.0041 (12)	0.0004 (10)	−0.0041 (12)
C19	0.0384 (13)	0.0465 (18)	0.0740 (16)	−0.0093 (11)	0.0039 (11)	0.0008 (13)
C20	0.0410 (13)	0.0383 (16)	0.0592 (14)	−0.0014 (11)	0.0054 (10)	0.0063 (11)

### Geometric parameters (Å, º)

**Table:**

S1—C5	1.774 (2)	C6—H6B	0.97
S1—C6	1.800 (2)	C7—C8	1.487 (3)
S2—C5	1.653 (2)	C8—C9	1.387 (3)
O1—C1	1.401 (3)	C8—C13	1.394 (3)
O1—C4	1.407 (3)	C9—C10	1.374 (3)
O2—C7	1.214 (2)	C9—H9	0.93
O3—C14	1.354 (3)	C10—C11	1.381 (3)
O3—C12	1.410 (2)	C10—H10	0.93
O4—C14	1.191 (3)	C11—C12	1.367 (3)
N1—C5	1.338 (3)	C11—H11	0.93
N1—C3	1.472 (3)	C12—C13	1.374 (3)
N1—C2	1.480 (3)	C13—H13	0.93
C1—C2	1.493 (4)	C14—C15	1.479 (3)
C1—H1A	0.97	C15—C16	1.382 (3)
C1—H1B	0.97	C15—C20	1.383 (3)
C2—H2A	0.97	C16—C17	1.378 (3)
C2—H2B	0.97	C16—H16	0.93
C3—C4	1.474 (4)	C17—C18	1.370 (3)
C3—H3A	0.97	C17—H17	0.93
C3—H3B	0.97	C18—C19	1.371 (3)
C4—H4A	0.97	C18—H18	0.93
C4—H4B	0.97	C19—C20	1.382 (3)
C6—C7	1.507 (3)	C19—H19	0.93
C6—H6A	0.97	C20—H20	0.93
			
C5—S1—C6	101.33 (10)	C8—C7—C6	118.02 (18)
C1—O1—C4	111.06 (19)	C9—C8—C13	119.1 (2)
C14—O3—C12	116.89 (17)	C9—C8—C7	118.78 (19)
C5—N1—C3	122.22 (19)	C13—C8—C7	122.1 (2)
C5—N1—C2	125.35 (19)	C10—C9—C8	120.6 (2)
C3—N1—C2	111.70 (18)	C10—C9—H9	119.7
O1—C1—C2	112.8 (2)	C8—C9—H9	119.7
O1—C1—H1A	109	C9—C10—C11	120.4 (2)
C2—C1—H1A	109	C9—C10—H10	119.8
O1—C1—H1B	109	C11—C10—H10	119.8
C2—C1—H1B	109	C12—C11—C10	118.6 (2)
H1A—C1—H1B	107.8	C12—C11—H11	120.7
N1—C2—C1	109.3 (2)	C10—C11—H11	120.7
N1—C2—H2A	109.8	C11—C12—C13	122.4 (2)
C1—C2—H2A	109.8	C11—C12—O3	120.3 (2)
N1—C2—H2B	109.8	C13—C12—O3	117.27 (19)
C1—C2—H2B	109.8	C12—C13—C8	118.9 (2)
H2A—C2—H2B	108.3	C12—C13—H13	120.6
N1—C3—C4	110.6 (2)	C8—C13—H13	120.6
N1—C3—H3A	109.5	O4—C14—O3	122.2 (2)
C4—C3—H3A	109.5	O4—C14—C15	125.4 (2)
N1—C3—H3B	109.5	O3—C14—C15	112.34 (19)
C4—C3—H3B	109.5	C16—C15—C20	119.5 (2)
H3A—C3—H3B	108.1	C16—C15—C14	117.8 (2)
O1—C4—C3	113.0 (2)	C20—C15—C14	122.6 (2)
O1—C4—H4A	109	C17—C16—C15	120.3 (2)
C3—C4—H4A	109	C17—C16—H16	119.8
O1—C4—H4B	109	C15—C16—H16	119.8
C3—C4—H4B	109	C18—C17—C16	119.8 (2)
H4A—C4—H4B	107.8	C18—C17—H17	120.1
N1—C5—S2	124.08 (17)	C16—C17—H17	120.1
N1—C5—S1	113.60 (15)	C17—C18—C19	120.3 (2)
S2—C5—S1	122.32 (13)	C17—C18—H18	119.8
C7—C6—S1	108.88 (15)	C19—C18—H18	119.8
C7—C6—H6A	109.9	C18—C19—C20	120.3 (2)
S1—C6—H6A	109.9	C18—C19—H19	119.9
C7—C6—H6B	109.9	C20—C19—H19	119.9
S1—C6—H6B	109.9	C19—C20—C15	119.7 (2)
H6A—C6—H6B	108.3	C19—C20—H20	120.2
O2—C7—C8	121.2 (2)	C15—C20—H20	120.2
O2—C7—C6	120.8 (2)		

### Hydrogen-bond geometry (Å, º)

Cg is the centroid of the C15–C20 ring.

**Table:**

*D*—H···*A*	*D*—H	H···*A*	*D*···*A*	*D*—H···*A*
C2—H2*B*···S1	0.97	2.41	2.938 (2)	114
C3—H3*A*···S2	0.97	2.56	3.052 (5)	111
C13—H13···O4^i^	0.93	2.43	3.224 (3)	143
C11—H11···*Cg*^ii^	0.93	2.88	3.629 (2)	138

Symmetry codes: (i) *x*, *y*+1, *z*; (ii) −*x*+1, −*y*, −*z*+1.
